# Identification of QTL Associated with Nitrogen Uptake and Nitrogen Use Efficiency Using High Throughput Genotyped CSSLs in Rice (*Oryza sativa* L.)

**DOI:** 10.3389/fpls.2017.01166

**Published:** 2017-07-11

**Authors:** Yong Zhou, Yajun Tao, Dongnan Tang, Jun Wang, Jun Zhong, Yi Wang, Qiumei Yuan, Xiaofeng Yu, Yan Zhang, Yulong Wang, Guohua Liang, Guichun Dong

**Affiliations:** ^1^Jiangsu Key Laboratory of Crop Genetics and Physiology/Co-Innovation Center for Modern Production Technology of Grain Crops, Key Laboratory of Plant Functional Genomics of the Ministry of Education, Yangzhou University Yangzhou, China; ^2^Institute of Food Crops, Jiangsu High Quality Rice Research and Development Center, Nanjing Branch of China National Center for Rice Improvement, Jiangsu Academy of Agricultural Sciences Nanjing, China

**Keywords:** rice, nitrogen uptake, nitrogen use efficiency, QTL mapping, CSSLs

## Abstract

Nitrogen (N) availability is a major factor limiting crop growth and development. Identification of quantitative trait loci (QTL) for N uptake (NUP) and N use efficiency (NUE) can provide useful information regarding the genetic basis of these traits and their associated effects on yield production. In this study, a set of high throughput genotyped chromosome segment substitution lines (CSSLs) derived from a cross between recipient 9311 and donor Nipponbare were used to identify QTL for rice NUP and NUE. Using high throughput sequencing, each CSSL were genotyped and an ultra-high-quality physical map was constructed. A total of 13 QTL, seven for NUP and six for NUE, were identified in plants under hydroponic culture with all nutrients supplied in sufficient quantities. The proportion of phenotypic variation explained by these QTL for NUP and NUE ranged from 3.16–13.99% and 3.76–12.34%, respectively. We also identified several QTL for biomass yield (BY) and grain yield (GY), which were responsible for 3.21–45.54% and 6.28–7.31%, respectively, of observed phenotypic variation. GY were significantly positively correlated with NUP and NUE, with NUP more closely correlated than NUE. Our results contribute information to NUP and NUE improvement in rice.

## Introduction

The world’s population will reach 9 billion by 2050 (Gregory and George, 2011). Food shortages are becoming a serious global problem. To guarantee global food security for future generations in view of this population explosion, the estimated annual increase in agricultural productivity needs to be raised to 3% from the current 2% (von Braun, 2010). Nitrogen (N), an essential element for crop growth, is considered to be the main factor limiting crop productivity, second only to water deficiency. In most agricultural regions, crop production is highly dependent on supply of exogenous N fertilizer (Kraiser et al., 2011). To satisfy the food requirements of an increasing population, the amount of synthetic N applied to crops has risen dramatically, from 12 to 104 terrogram per year (Tg year^-1^) over the last 40 years (Mulvaney et al., 2009; Godfray et al., 2010). Much of the N applied to the soil is lost to the atmosphere or leached into groundwater and other freshwater bodies, however, with an average of only 30–50% of total applied N actually harvested in grains. N fertilizer input is essential to high crop yield, but excessive use of N fertilizer causes many pollution such as air pollution and water pollution (Vance, 2001; Hirel et al., 2007; Vitousek et al., 2009; Godfray et al., 2010; Liu et al., 2010, 2013). The development of crops with high N utilization ability is therefore urgently needed. N utilization can be subdivided into two processes: N uptake (the ability of the plant to remove N from the soil as nitrate and ammonium ions, NUP) and N utilization (efficiency of N use to produce grain yield, NUE). The urgent need for advances in agricultural research and technology, especially in the aspect of N utilization, has raised strict requirements that we must develop sustainable agricultural methods to increase food yield (Zeigler and Mohanty, 2010).

Rice (*Oryza sativa* L.) is an important food and feeds half of the world’s population (Zhou et al., 2009). As a fertilizer, N is widely used in the whole stage of rice growth and development (Cassman et al., 1998). In China, N fertilizer is excessively used and accounting for 35% of global N fertilizer consumption (FAO, 2004), 7% of which is applied to irrigated rice (Peng et al., 2006). In recent years, N fertilizer application rates in many regions of China have been significantly higher than the world average. The average amount of N fertilizer applied annually in China is 220 kg ha^-1^, with rates as high as 314 kg ha^-1^ over a single rice season (Shen and Zhang, 2006; Zeng et al., 2012). Although N fertilizer application is one of the major expenses incurred by Chinese rice farmers, rice NUE is less than 30–35%, resulting in N losses over 50% (Peng et al., 2002, 2006; Shen and Zhang, 2006). Reducing N input is beneficial not only to farmers but also to the environment. Consequently, the introduction of rice varieties with high NUP and NUE is now an objective of many rice breeding programs (Dong et al., 2006), which necessitates a better understanding of the genetic basis of NUP and NUE in rice.

Quantitative trait locus (QTL) mapping can reveal chromosomal locations of unknown genes that influence quantitative variation of complex traits such as NUP and NUE. Several studies have been carried out to map QTL for NUE in rice. Using 98 backcross inbred lines (BILs) developed from Nipponbare and Kasalash, QTL associated with rice NUE were firstly mapped (Obara et al., 2001). One QTL, *qNUEP-6*, controlling NUE on chromosome 6, was identified in a recombinant inbred line (RIL) population of Zhenshan 97 and Minghui 63 (Shan et al., 2005). Under three N levels, a major QTL associated with NUE on chromosome 3 was detected in a doubled haploid (DH) population from IR64 and Azucena (Senthilvel et al., 2008). Under low N conditions, one QTL for NUE, *pnue9*, was identified on chromosome 9 using a RIL population from Dasanbyeo and TR22183 (Cho et al., 2007). Dozens of QTL for various traits correlated with NUE were detected in two *japonica* × *japonica* RILs (Li et al., 2010). Four and six QTL for rice NUE were identified in 2006 and 2007, respectively, using 127 RILs derived from Zhenshan 97 and Minghui 63 (Wei et al., 2011). Reports have also appeared on QTL analysis of NUP or N content (Ishimaru et al., 2001; Ju et al., 2006; Cho et al., 2007; Piao et al., 2009), low N tolerance (Lian et al., 2005; Wei et al., 2012), and glutamine synthetase content (Obara et al., 2001, 2004). Despite these advances, the genetic control of NUP and NUE in rice is still not well-understood.

Our study was designed to precisely characterize more QTL for rice NUP and NUE under hydroponic conditions using a population of chromosome segment substitution lines (CSSLs). These results provide useful information for further dissection of the genetic basis of NUE and NUP, and should facilitate development of rice varieties with better nitrogen uptake and nitrogen use efficiency.

## Materials and Methods

### Materials

As described previously, to uncover the genetic basis of rice important agronomic traits, a population consisting of 128 CSSLs, developed from a cross between *japonica* cultivar Nipponbare as the donor parent and *indica* cultivar 9311 as the recurrent parent, was developed (Xu et al., 2010). Based on high throughput sequencing, an ultra-high-quality physical map of the CSSLs was constructed (Xu et al., 2010). According to the physical map, the 128 CSSLs totally carried 142 substituted chromosome segments, which ranged from 0.65 to 22.3 Mb with an average of 6.21 Mb. In this study, these 128 CSSLs were employed for QTL mapping of rice NUP, NUE and other related traits.

### Hydroponic Culture

Chromosome segment substitution lines and their parents were grown under hydroponic conditions at Yangzhou University (latitude 32°24′ N, longitude 119°26′ E), Yangzhou City, China. All rice seedlings were solution-cultured in eight 5.72 m^3^ (8.8 m × 1.3 m × 0.5 m) concrete ponds connected to one another at the bottom by iron tubes. The ponds were covered with concrete planks (135.0 cm × 16.7 cm × 2.5 cm) containing 14 holes (4 cm in diameter) for seedling fixation (Dong et al., 2011). Plump seeds were surface-sterilized in a 2.5% NaClO solution for 15 min, and then washed three times with distilled water. In an illumination incubator, seeds were germinated at 30°C and transplanted into the holes after 30 days. 56 seedlings for each line were planted in a randomized complete block design with two replications. The nutrient solution was formulated with a N concentration of 7 mg L^-1^, as described previously (Dong et al., 2006, 2011).

When transplanting, the nutrient solution was poured into the ponds and refreshed every 10 days. In order to maintain pH between 5.5 and 6.5, we added diluted H_2_SO_4_. We also improved O_2_ supply and used a pump to keeping the solution cycling continuously.

### Evaluation of Phenotypes

We measured NUP, NUE, biomass yield (BY), and grain yield (GY) at plant maturity. After deactivating enzymes at 105°C for 10 min, each plant portion (including aboveground organs and roots) was dried at 75°C to constant weight, and then weighed to obtain BY (g m^-2^). For GY (g m^-2^), total grains were stored and dried at room temperature for at least 1 month before weighing.

N content of grains (N_grain_), shoots (N_shoot_), and roots (N_root_) was estimated in each line by the Kjeldahl method, and plant N content (N_plant_) was calculated as N_grain_ + N_shoot_ + N_root_. Total N uptake ability (NUP) was then calculated as N_plant_ = (N_grain_ + N_shoot_ + N_root_)/S (g m^-2^). S denotes the sampled area. NUE was calculated as GY/N_plant_ (g g^-1^).

### Statistical and QTL Analyses

Phenotypic data were analyzed using SPSS statistical software, and frequency distributions were plotted using Sigma Plot 10.0. QTL analysis was conducted as described previously (Xu et al., 2010). The following multiple linear model was used in the SAS software package:

$$y_{i}=b_{0}+\overset{m}{\underset{k=1}{\Sigma }}b_{k}x_{ik}+e_{i}$$

where *y_i_*, *b*_0_ denotes the mean value of the *i*th CSSL line and the mean of overall population, respectively. *m* is the total number of bins in the whole genome, *b_k_* is the main effect associated with bin *k. x_ik_* = 1 or -1 corresponding to the donor parent and the recurrent parent bin, respectively. *e_i_* is the residual error. According to multiple linear regression, we estimated the contributions of each bin to phenotypic variation.

## Results

### Phenotypic Performance of CSSLs and Their Parents

A set of CSSLs and their parents (9311 and Nipponbare) under hydroponic culture conditions were investigated. Phenotypic values of NUP and NUE in the CSSLs and parents are listed in **Table 1**. The NUP of 9311 was 1.60-fold higher than that of Nipponbare, whereas the NUE was similar (28.60 ± 1.00 g g^-1^ vs. 27.74 ± 0.95 g g^-1^). 9311 is a high-yield *indica* variety developed in the 1990s, and Nipponbare is a low-yield *japonica* variety developed in the 1950s. We also compared BY and GY of these two parents. BY and GY of 9311 were 1.39- and 1.65-fold higher, respectively, than those of Nipponbare. These data indicate that the higher yield of 9311 is due to increased NUP rather than NUE.

**Table 1:** Mean values and ranges of nitrogen uptake (NUP), nitrogen use efficiency (NUE), biomass yield (BY), and grain yield (GY) in rice CSSLs and their parents.

Traits	Parents (mean ± SD)		CSSLs	
		
	9311	Nipponbare	Mean ± SD	Range
NUP	24.02 ± 1.62	15.00 ± 0.64	29.71 ± 4.40	14.88–44.67
NUE	28.60 ± 1.00	27.74 ± 0.95	24.53 ± 4.45	10.84–32.50
BY	1628.26 ± 105.56	1171.70 ± 47.24	1913.92 ± 250.63	984.80–2893.83
GY	686.83 ± 58.19	416.11 ± 31.82	719.90 ± 132.74	322.12–1009.55


**FIGURE 1:**
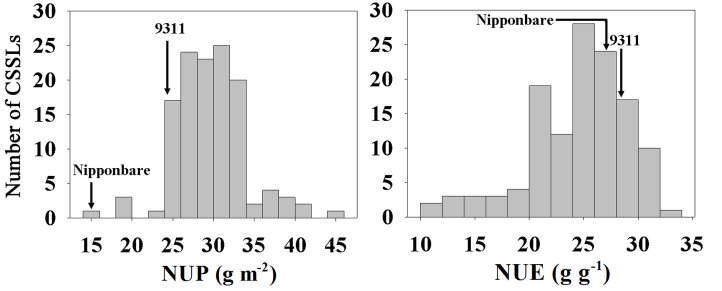
N uptake (NUP) and NUE distributions in the CSSLs under hydroponic culture.

In the CSSL population, extensive variation, and normal distributions were observed for NUP and NUE, consistent with the characteristics of quantitative traits (**Figure 1**). NUP, BY and GY in the CSSL population were all closer to those of 9311 (**Table 1**), as 9311 was the recurrent parent.

### Relationship between Yield Production and N Utilization

**FIGURE 2:**
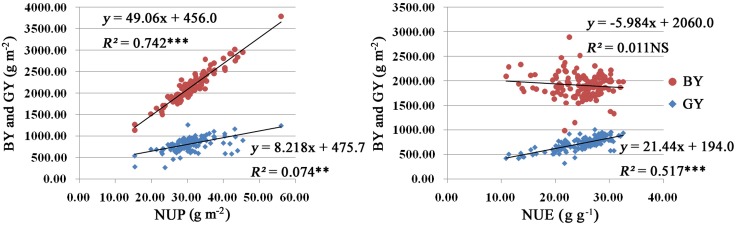
Results of correlation analysis among NUP, NUE, BY, and GY in rice plants under hydroponic culture. The linear regression equation model used and *R^2^* are listed at the top right. Red and blue indicate BY and GY, respectively. Significance levels are as follows: ^∗^*p* < 0.05; ^∗∗^*p* < 0.01; ^∗∗∗^*p* < 0.001 level; NS, not significant.

We carried out a correlation analysis among NUP, NUE, BY, and GY. As shown in **Figure 2**, NUP was significantly positively correlated with BY (*R^2^* = 0.742). Similar relationships were found between NUP and GY, with *R^2^* values of 0.074 (**Figure 2**). Thus, the increased NUP could led to an increased BY and GY. NUP was more closely correlated with BY than with GY.

N use efficiency displayed significant positive correlations with GY, with *R^2^* values of 0.517 (**Figure 2**). However, there was no significance correlation between NUE and BY.

### QTL Mapping

#### N Uptake

We detected seven putative QTL controlling NUP: *qNUP2.1*, *qNUP3.1*, *qNUP6.1*, *qNUP8.1*, *qNUP10.1*, *qNUP11.1*, and *qNUP11.2*. These QTL were found on chromosomes 2, 3, 6, 8, 10, and 11. Contributions of these QTL to observed phenotypic variance were 3.83, 4.75, 11.86, 13.99, 9.80, 3.16, and 4.30%, respectively. The QTL with the largest effect was mapped to X_278_ and occupied to the physical position of 2797908–3336084 bp (**Table 2**).

**Table 2:** Quantitative trait loci mapping associated with NUP and NUE in rice plants under hydroponic culture.

Traits	Bins	QTL	Interval (bp)	Interval size (bp)	Chromosome	Partial R-square	*F*-Value
NUP	X_107_	*qNUP2.1*	36017977–36777825	759848	2	3.83%	8.86
	X_141_	*qNUP3.1*	25056241–25069454	13213	3	4.75%	9.64
	X_233_	*qNUP6.1*	7814673–9668398	1853725	6	11.86%	19.68
	X_278_	*qNUP8.1*	2797908–3336084	538176	8	13.99%	20.16
	X_362_	*qNUP10.1*	22335288–22517954	182666	10	9.80%	18.57
	X_380_	*qNUP11.1*	19120157–19494142	373985	11	3.16%	7.73
	X_387_	*qNUP11.2*	25559185–26317711	758526	11	4.30%	9.34
NUE	X_100_	*qNUE2.1*	31531953–32386052	854099	2	3.98%	6.96
	X_176_	*qNUE4.1*	23285463–23315504	30041	4	4.40%	7.34
	X_231_	*qNUE6.1*	6517443–6942384	424941	6	12.34%	17.46
	X_234_	*qNUE6.2*	9668398–9927733	259335	6	4.79%	7.59
	X_343_	*qNUE10.1*	17355105–17376675	21570	10	3.76%	6.90
	X_354_	*qNUE10.2*	20364788–20798359	433571	10	5.87%	8.84


#### N Use Efficiency

A total of six QTL were detected for NUE. These QTL were *qNUE2.1*, *qNUE4.1*, *qNUE6.1*, *qNUE6.2*, *qNUE10.1*, and *qNUE10.2*, which were located in X_100_ on chromosome 2, X_176_ on chromosome 4, X_231_ on chromosome 6, X_234_ on chromosome 6 X_343_ on chromosome 10 and X_354_ on chromosome 10, respectively. *qNUE6.1*, is the QTL having the largest contribution to the phenotypic variance with its contribution of 12.34% (**Table 2**).

#### Grain Yield

Two putative QTL for GY were detected. These QTL, with their locations and phenotypic contributions, were as follows: *qGY6.1* in X_231_ on chromosome 6 (6.28%), and *qGY8.1* in X_277_ on chromosome 8 (7.31%) (**Table 3**).

**Table 3:** Quantitative trait loci mapping associated with GY and BY in rice plants under hydroponic culture.

Traits	Bins	QTL	Interval (bp)	Interval size (bp)	Chromosome	Partial R-square	*F*-Value
GY	X_231_	*qGY6.1*	6517443–6942384	424941	6	6.28%	8.95
	X_277_	*qGY8.1*	2492172–2797908	305736	8	7.31%	9.78
BY	X_37_	*qBY1.1*	40660285–40695764	35479	1	11.47%	19.22
	X_107_	*qBY2.1*	36017977–36777825	759848	2	3.21%	7.18
	X_108_	*qBY2.2*	36777825–36823111	45286	2	3.98%	9.55
	X_130_	*qBY3.1*	12844058–13297480	453422	3	45.54%	9.19
	X_233_	*qBY6.1*	7814673–9668398	1853725	6	4.60%	8.71
	X_278_	*qBY8.1*	2797908–3336084	538176	8	15.10%	22.05
	X_362_	*qBY10.1*	22335288–22517954	182666	10	5.01%	8.93
	X_387_	*qBY11.1*	25559185–26317711	758526	11	3.34%	7.10


#### Biomass Yield

In total, eight QTL (*qBY1.1*, *qBY2.1*, *qBY2.2*, *qBY3.1, qBY6.1, qBY8.1*, *qBY10.1*, and *qBY11.1*) for BY were detected, which were located in X_37_ on chromosome 1, X_107_ on chromosome 2, X_108_ on chromosome 2, X_130_ on chromosome 3, X_233_ on chromosome 6, X_278_ on chromosome 8, X_362_ on chromosome 10 and X_387_ on chromosome 11, respectively. These QTL explained 11.47, 3.21, 3.98, 45.54, 4.60, 15.10, 5.01, and 3.34%, respectively of the phenotypic variance. The QTL with the largest effect was mapped to X_130_ (**Table 3**).

From the above analysis, we can find that several QTL show pleiotropic effect among traits. For example, the location X_278_ occupied the physical position of 2797908–3336084 bp comprises a 538176 bp region on chromosome 8, which simultaneously controlled NUP and BY. In X_107_, X_233_, X_278_, X_362,_ and X_387_ on chromosome 2, 6, 8, and 11, respectively, the location also controlled NUP and BY simultaneously. Both *qNUE6.1* and *qGY6.1* were detected in X_231_ on chromosome 6. These results implied that these QTL may have potential value in rice breeding.

## Discussion

N uptake and NUE improvement is becoming a major objective of modern rice breeding programs. The advent of modern breeding technologies has accelerated the pace of agricultural improvements. These new breeding methods not only affect crop yield, but also help meet the challenges associated with environmental sustainability, food supply, and fossil fuel replacement. A better understanding of the genetic basis of NUP and NUE, especially the relationship between these two traits and grain yield in rice, could be very important for food security and environmental benefits. Because of low heritability, field selection of NUP and NUE is always disturbed. Marker-assisted selection (MAS) enables the development of varieties with multiple target traits controlled by small-effect QTL/genes. Once the genes associated with agricultural and economical interest have been found, MAS can integrate this biological and genomic information into traditional crop breeding programs to greatly improve breeding efficiency. Most successful MAS breeding research, however, has been conducted on heading date (Takeuchi et al., 2006) and resistance to diseases or insects conferred by major genes (Fjellstrom et al., 2004, 2006; Jia et al., 2004; Hayashi et al., 2006). Few studies have addressed the improvement of complex quantitative traits including NUP and NUE; this situation is possibly due to lack of information on QTL epistasis, QTL × environment interaction (QEI) effects, gene action of QTL, and markers closely linked to target QTL.

Most agronomic traits, including NUP and NUE, are typical quantitative traits controlled by several genes, or QTL. Multiple QTL associated with NUP and NUE have been assigned to linkage maps using different mapping populations. Because of the noise of genetic background, F_2_, DHs, and RILs are rarely used for QTL fine mapping or cloning (Yamamoto et al., 2000). In this study, we used CSSLs as a mapping population to detect QTL for NUP and NUE under hydroponic conditions. CSSLs have the similar genetic backgrounds, except for the substituted segments, as the recurrent parent, making it possible to divide QTL into single Mendelian factors.

In the CSSLs, variation ranges of the target traits are large (**Table 1**). NUP, BY and GY in the CSSL population were closer to those of 9311, as 9311 was the recurrent parent. We also noticed that the average NUP of CSSLs was much higher than that of 9311. However, the average NUE of CSSLs was much lower. So, Nipponbare may carry multiple quantitative loci promoting rice nitrogen uptake. Actually, the NUP of Nipponbare itself was so low. A possible explanation is that gene interaction or antagonism between different QTL for NUP may exist in Nipponbare.

Because NUP and NUE both had large effects on rice grain yield, we also carried out QTL mapping for GY and BY using these CSSLs. We identified 23 QTL: 7 for NUP, 6 for NUE, 8 for BY, and 2 for GY under hydroponic culture. These QTL were located in 17 bins, with five QTL in Bins smaller than 50 kb: *qNUP3.1*, *qNUE4.1*, *qNUE10.1*, *qBY1.1*, and *qBY2.2*. These five QTL, with their small marker interval distances, are ideal for fine mapping, and their use in future studies should reduce time and labor costs.

**FIGURE 3:**
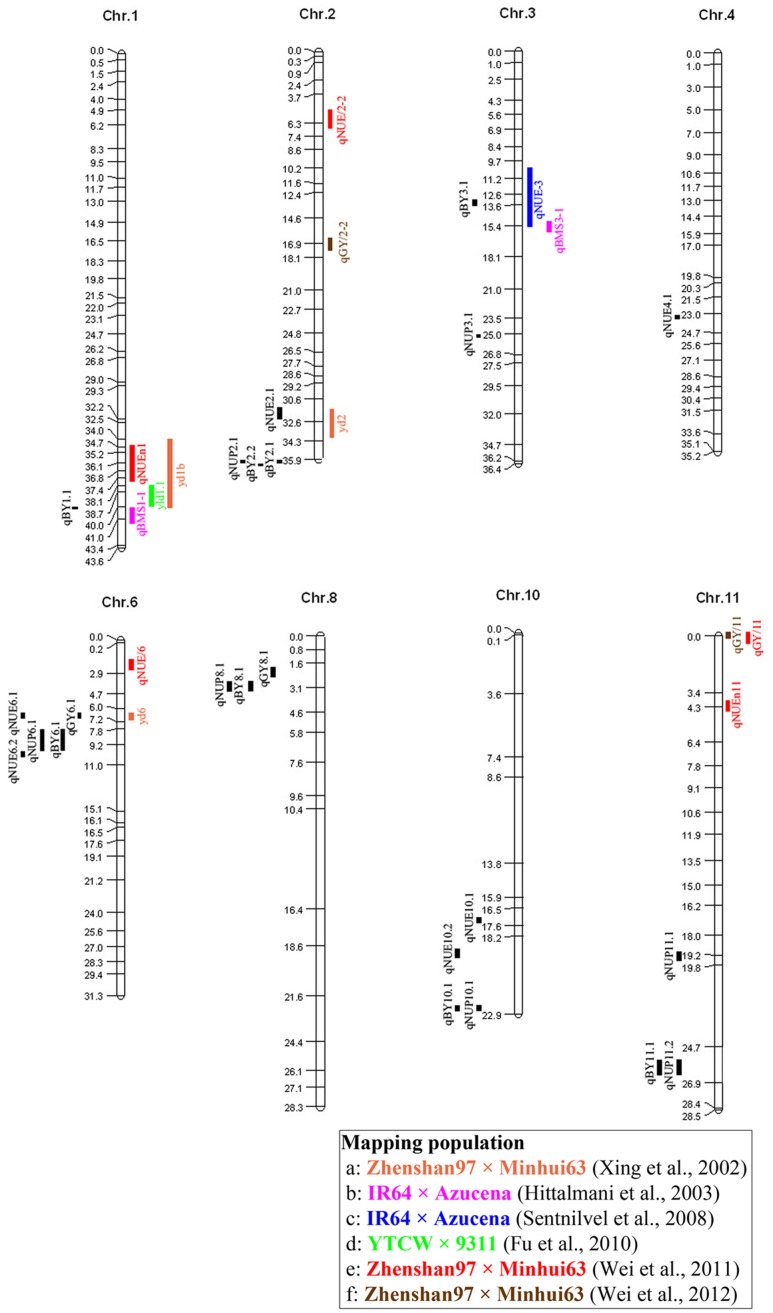
Rice molecular linkage map (without marker names) showing bin allocation of QTL detected in this study and six other mapping populations (a–f) evaluated for NUP, NUE, GY, and BY. QTL identified in this study and QTL identified in the six other mapping populations are labeled using acronyms on left and right sides, respectively, of chromosomes. The six populations (a–f) are labeled with different colors in accordance with corresponding acronyms for related traits.

We found that regions controlling BY in our study contained QTL for NUE detected in previous studies (**Figure 3**). For example, *qBY1.1*, which was detected in X_37_ and closed to the QTL *qBMS1-1* and *yld1.1*, responsible for biomass per plant and grain yield per plant, respectively (Hittalmani et al., 2003; Fu et al., 2010), and was also close to the QTL, *qNUEn1*, for NUE detected in RILs from Zhenshan 97 and Minghui 63 (Wei et al., 2011). *qBY3.1* on X_130_ with the region 12844058–13297480 bp close to *qNUE-3* detected in IR64 and Azucena (Senthilvel et al., 2008). Additionally, this QTL explained 45.54% of the phenotypic variance, which should be further mapped to make it useful in rice breeding. Similarly, QTL for NUE and NUP detected in our study were located in the vicinity of QTL associated with GY in previous studies (**Figure 3**). For example, *qNUE2.1* was detected in bin X_100_, the region encompassing positions 31531953–32386052, near the region where a QTL controlling GY in RILs from Zhenshan 97 and Minghui 63 has been detected (Xing et al., 2002). *qNUE6.1* detected in bin X_231_, the region encompassing positions 6517443–6942384, was located close to the QTL *yd6* detected in Zhenshan 97 and Minghui 63 has been detected (Xing et al., 2002). However, hardly a region controlling NUE or NUP were both detected in our or previous studies. One explanation is that QTL associated with NUE and NUP were highly susceptible to environment.

Our correlation analysis results suggest that while both NUP and NUE have large effects on rice grain yield, NUP is more closely correlated with grain yield than NUE. In our previous studies (Dong et al., 2006, 2011), we reached similar conclusions based on NUP and NUE data from different varieties. It can thus be assumed that increasing NUP rather than NUE would be a more effective strategy for improving grain yield.

In this study, we identified 7 QTL for NUP and 6 QTL for NUE. Based on this information, we can pyramid high NUP and NUE loci using MAS to develop high-yield rice varieties requiring low N fertilizer input.

## Author Contributions

YoZ conducted the statistical analysis and paper drafting; YT performed the experiments and data analysis; DT conducted the statistical analysis; JW revised the manuscript; JZ performed the experiments; YiW performed the experiments; QY performed the experiments; XY performed the experiments; YaZ performed the experiments; YuW performed the experiments; GL supervised the work and finalized the manuscript; GD designed the experiments and supervised the work.

## Conflict of Interest Statement

The authors declare that the research was conducted in the absence of any commercial or financial relationships that could be construed as a potential conflict of interest.
